# ARIA 2016: Care pathways implementing emerging technologies for predictive medicine in rhinitis and asthma across the life cycle

**DOI:** 10.1186/s13601-016-0137-4

**Published:** 2016-12-30

**Authors:** J. Bousquet, P. W. Hellings, I. Agache, A. Bedbrook, C. Bachert, K. C. Bergmann, M. Bewick, C. Bindslev-Jensen, S. Bosnic-Anticevitch, C. Bucca, D. P. Caimmi, P. A. M. Camargos, G. W. Canonica, T. Casale, N. H. Chavannes, A. A. Cruz, G. De Carlo, R. Dahl, P. Demoly, P. Devillier, J. Fonseca, W. J. Fokkens, N. A. Guldemond, T. Haahtela, M. Illario, J. Just, T. Keil, L. Klimek, P. Kuna, D. Larenas-Linnemann, M. Morais-Almeida, J. Mullol, R. Murray, R. Naclerio, R. E. O’Hehir, N. G. Papadopoulos, R. Pawankar, P. Potter, D. Ryan, B. Samolinski, H. J. Schunemann, A. Sheikh, F. E. R. Simons, C. Stellato, A. Todo-Bom, P. V. Tomazic, A. Valiulis, E. Valovirta, M. T. Ventura, M. Wickman, I. Young, A. Yorgancioglu, T. Zuberbier, W. Aberer, C. A. Akdis, M. Akdis, I. Annesi-Maesano, J. Ankri, I. J. Ansotegui, J. M. Anto, S. Arnavielhe, A. Asarnoj, H. Arshad, F. Avolio, I. Baiardini, C. Barbara, M. Barbagallo, E. D. Bateman, B. Beghé, E. H. Bel, K. S. Bennoor, M. Benson, A. Z. Białoszewski, T. Bieber, L. Bjermer, H. Blain, F. Blasi, A. L. Boner, M. Bonini, S. Bonini, I. Bosse, J. Bouchard, L. P. Boulet, R. Bourret, P. J. Bousquet, F. Braido, A. H. Briggs, C. E. Brightling, J. Brozek, R. Buhl, C. Bunu, E. Burte, A. Bush, F. Caballero-Fonseca, M. A. Calderon, T. Camuzat, V. Cardona, P. Carreiro-Martins, A. M. Carriazo, K. H. Carlsen, W. Carr, A. M. Cepeda Sarabia, M. Cesari, L. Chatzi, R. Chiron, T. Chivato, E. Chkhartishvili, A. G. Chuchalin, K. F. Chung, G. Ciprandi, J. Correia de Sousa, L. Cox, G. Crooks, A. Custovic, S. E. Dahlen, U. Darsow, T. Dedeu, D. Deleanu, J. A. Denburg, G. De Vries, A. Didier, A. T. Dinh-Xuan, D. Dokic, H. Douagui, G. Dray, R. Dubakiene, S. R. Durham, G. Du Toit, M. S. Dykewicz, P. Eklund, Y. El-Gamal, E. Ellers, R. Emuzyte, J. Farrell, A. Fink Wagner, A. Fiocchi, M. Fletcher, F. Forastiere, M. Gaga, A. Gamkrelidze, B. Gemicioğlu, J. E. Gereda, R. Gerth van Wick, S. González Diaz, I. Grisle, L. Grouse, Z. Gutter, M. A. Guzmán, B. Hellquist-Dahl, J. Heinrich, F. Horak, J. O’. B. Hourihane, M. Humbert, M. Hyland, G. Iaccarino, E. J. Jares, C. Jeandel, S. L. Johnston, G. Joos, O. Jonquet, K. S. Jung, M. Jutel, I. Kaidashev, M. Khaitov, O. Kalayci, A. F. Kalyoncu, P. Kardas, P. K. Keith, M. Kerkhof, H. A. M. Kerstjens, N. Khaltaev, M. Kogevinas, V. Kolek, G. H. Koppelman, M. L. Kowalski, M. Kuitunen, I. Kull, V. Kvedariene, B. Lambrecht, S. Lau, D. Laune, L. T. T. Le, P. Lieberman, B. Lipworth, J. Li, K. C. Lodrup Carlsen, R. Louis, C. Lupinek, W. MacNee, Y. Magar, A. Magnan, B. Mahboub, D. Maier, I. Majer, J. Malva, P. Manning, E. De Manuel Keenoy, G. D. Marshall, M. R. Masjedi, E. Mathieu-Dupas, M. Maurer, S. Mavale-Manuel, E. Melén, E. Melo-Gomes, E. O. Meltzer, J. Mercier, H. Merk, N. Miculinic, F. Mihaltan, B. Milenkovic, J. Millot-Keurinck, Y. Mohammad, I. Momas, R. Mösges, A. Muraro, L. Namazova-Baranova, R. Nadif, H. Neffen, K. Nekam, A. Nieto, B. Niggemann, L. Nogueira-Silva, M. Nogues, T. D. Nyembue, K. Ohta, Y. Okamoto, K. Okubo, M. Olive-Elias, S. Ouedraogo, P. Paggiaro, I. Pali-Schöll, S. Palkonen, P. Panzner, A. Papi, H. S. Park, G. Passalacqua, S. Pedersen, A. M. Pereira, O. Pfaar, R. Picard, B. Pigearias, I. Pin, D. Plavec, W. Pohl, T. A. Popov, F. Portejoie, D. Postma, L. K. Poulsen, D. Price, K. F. Rabe, F. Raciborski, G. Roberts, C. Robalo-Cordeiro, F. Rodenas, L. Rodriguez-Mañas, C. Rolland, M. Roman Rodriguez, A. Romano, J. Rosado-Pinto, N. Rosario, M. Rottem, M. Sanchez-Borges, J. Sastre-Dominguez, G. K. Scadding, N. Scichilone, P. Schmid-Grendelmeier, E. Serrano, M. Shields, V. Siroux, J. C. Sisul, I. Skrindo, H. A. Smit, D. Solé, T. Sooronbaev, O. Spranger, R. Stelmach, P. J. Sterk, T. Strandberg, J. Sunyer, C. Thijs, M. Triggiani, R. Valenta, A. Valero, M. van Eerd, E. van Ganse, M. van Hague, O. Vandenplas, L. L. Varona, B. Vellas, G. Vezzani, T. Vazankari, G. Viegi, T. Vontetsianos, M. Wagenmann, S. Walker, D. Y. Wang, U. Wahn, T. Werfel, B. Whalley, D. M. Williams, S. Williams, N. Wilson, J. Wright, B. P. Yawn, P. K. Yiallouros, O. M. Yusuf, A. Zaidi, H. J. Zar, M. E. Zernotti, L. Zhang, N. Zhong, M. Zidarn

**Affiliations:** 1 Montpellier University Hospital, Montpellier, France; 2 MACVIA-France, Contre les MAladies Chroniques pour un VIeillissement Actif en France, European Innovation Partnership on Active and Healthy Ageing Reference Site, Montpellier, France; 3 INSERM, U1168, Ageing and Chronic Diseases Epidemiological and Public Health Approaches, 94800 Villejuif, France; 4 Laboratory of Clinical Immunology, Department of Microbiology and Immunology, KU Leuven, Louvain, Belgium; 5 Transylvania University Brasov, Brasov, Romania; 6 Upper Airways Research Laboratory, ENT Department, Ghent University Hospital, Ghent, Belgium; 7 Allergy-Centre-Charité, Department of Dermatology and Allergy, Charité - Universitätsmedizin Berlin, Berlin, Germany; 8 Global Allergy and Asthma European Network (GA²LEN), Berlin, Germany; 9 iQ4U Consultants Ltd, London, UK; 10 Department of Dermatology and Allergy Centre, Odense University Hospital, Odense, Denmark; 11 Woolcock Institute of Medical Research, University of Sydney and Sydney Local Health District, Glebe, NSW Australia; 12 University Pneumology Unit-AOU Molinette, Hospital City of Health and Science of Torino, Turin, Italy; 13 Department of Respiratory Diseases, Montpellier University Hospital, Montpellier, France; 14 Department of Pediatrics, Medical School, Federal University of Minas Gerais, Belo Horizonte, Brazil; 15 Asthma and Allergy Clinic, Humanitas University, Rozzano, Milan, Italy; 16 Division of Allergy/Immunology, University of South Florida, Tampa, FL USA; 17 Department of Public Health and Primary Care, Leiden University Medical Center, Leiden, The Netherlands; 18 ProAR – Nucleo de Excelencia em Asma, Federal University of Bahia, Salvador, Brazil; 19 GARD Executive Committee, Salvador, Bahia Brazil; 20 EFA European Federation of Allergy and Airways Diseases Patients’ Associations, Brussels, Belgium; 21 EPAR U707 INSERM, Paris, France; 22 EPAR UMR-S UPMC, Paris VI, Paris, France; 23 Laboratoire de Pharmacologie Respiratoire UPRES EA220, Hôpital Foch, Suresnes Université Versailles, Saint-Quentin, France; 24 Center for Research in Health Technologies and Information Systems - CINTESIS, Universidade do Porto, Porto, Portugal; 25 Allergy Unit, Instituto CUF Porto e Hospital CUF Porto, Porto, Portugal; 26 Health Information and Decision Sciences Department - CIDES, Faculdade de Medicina, Universidade do Porto, Rua Dr. Plácido da Costa, s/n, 4200-450 Porto, Portugal; 27 Department of Otorhinolaryngology, Academic Medical Centre, Amsterdam, The Netherlands; 28 Institute of Health Policy and Management IBMG, Erasmus University, Rotterdam, The Netherlands; 29 Skin and Allergy Hospital, Helsinki University Hospital, Helsinki, Finland; 30 Federico II University Hospital Naples (R&D and DISMET), Naples, Italy; 31 Allergology Department, Centre de l’Asthme et des Allergies, Hôpital d’Enfants Armand-Trousseau (APHP), Sorbonne Universités, UPMC Univ Paris 06, UMR_S 1136, Institut Pierre Louis d’Epidémiologie et de Santé Publique, Equipe EPAR, 75013 Paris, France; 32 Institute of Social Medicine, Epidemiology and Health Economics, Charité - Universitätsmedizin Berlin, Berlin, Germany; 33 Institute for Clinical Epidemiology and Biometry, University of Wuerzburg, Würzburg, Germany; 34 Center for Rhinology and Allergology, Wiesbaden, Germany; 35 Division of Internal Medicine, Asthma and Allergy, Barlicki University Hospital, Medical University of Lodz, Lodz, Poland; 36 Clínica de Alergia, Asma y Pediatría, Hospital Médica Sur, Mexico City, Mexico; 37 Allergy and Clinical Immunology Department, Hospital CUF-Descobertas, Lisbon, Portugal; 38 ENT Department, Hospital Clinic, Clinical and Experimental Respiratory Immunoallergy, IDIBAPS, CIBERES, Universitat de Barcelona, Barcelona, Catalonia Spain; 39 MedScript Ltd, Dundalk, County Louth, Ireland; 40 Section of Otolaryngology-Head and Neck Surgery, The University of Chicago Medical Center and The Pritzker School of Medicine, The University of Chicago, Chicago, IL USA; 41 Department of Allergy, Immunology and Respiratory Medicine, Alfred Hospital and Central Clinical School, Monash University, Melbourne, VIC Australia; 42 Department of Immunology, Monash University, Melbourne, VIC Australia; 43 Center for Pediatrics and Child Health, Institute of Human Development, Royal Manchester Children’s Hospital, University of Manchester, Manchester, UK; 44 Allergy Department, 2nd Pediatric Clinic, Athens General Children’s Hospital “P&A Kyriakou”, University of Athens, Athens, Greece; 45 Department of Pediatrics, Nippon Medical School, Tokyo, Japan; 46 Allergy Diagnostic and Clinical Research Unit, University of Cape Town Lung Institute, Cape Town, South Africa; 47 Woodbrook Medical Centre, Loughborough, UK; 48 Allergy and Respiratory Research Group, The University of Edinburgh, Edinburgh, UK; 49 Department of Prevention of Environmental Hazards and Allergology, Medical University of Warsaw, Warsaw, Poland; 50 Department of Clinical Epidemiology and Biostatistics, McMaster University, Hamilton, ON Canada; 51 Allergy and Respiratory Research Group, Centre for Population Health Sciences, The University of Edinburgh Medical School, Edinburgh, UK; 52 Department of Pediatrics and Child Health, Department of Immunology, Faculty of Medicine, University of Manitoba, Winnipeg, MB Canada; 53 Division of Allergy and Clinical Immunology, University of Salerno, Salerno, Italy; 54 Centre of Pneumology, Faculty of Medicine, University of Coimbra, Coimbra, Portugal; 55 Department of ENT, Medical University of Graz, Graz, Austria; 56 Clinic of Children’s Diseases, Faculty of Medicine, Vilnius University, Vilnius, Lithuania; 57 Public Health Institute, Vilnius University, Vilnius, Lithuania; 58 European Academy of Paediatrics (EAP/UEMS-SP), Brussels, Belgium; 59 Department of Lung Diseases and Clinical Allergology, University of Turku, Turku, Finland; 60 Allergy Clinic, Terveystalo, Turku, Finland; 61 Unit of Geriatric Immunoallergology, University of Bari Medical School, Bari, Italy; 62 Sachs’ Children and Youth Hospital, Södersjukhuset, Stockholm, Sweden; 63 Institute of Environmental Medicine, Karolinska Institutet, Stockholm, Sweden; 64 Queen’s University, Belfast, Northern Ireland, UK; 65 Department of Pulmonology, Celal Bayar University, Manisa, Turkey; 66 Department of Dermatology, Medical University of Graz, Graz, Austria; 67 Swiss Institute of Allergy and Asthma Research (SIAF), University of Zurich, Davos, Switzerland; 68 Department of Allergy and Immunology, Hospital Quirón Bizkaia, Erandio, Spain; 69 Barcelona Institute for Global Health (ISGlobal), Barcelona, Spain; 70 IMIM (Hospital del Mar Research Institute), Barcelona, Spain; 71 CIBER Epidemiología y Salud Pública (CIBERESP), Barcelona, Spain; 72 Universitat Pompeu Fabra (UPF), Barcelona, Spain; 73 Kyomed, Montpellier, France; 74 Clinical Immunology and Allergy Unit, Department of Medicine Solna, Karolinska Institutet, Stockholm, Sweden; 75 Department of Pediatric Pulmonology and Allergy, Astrid Lindgren Children’s Hospital, Karolinska University Hospital, Stockholm, Sweden; 76 David Hide Asthma and Allergy Research Centre, Isle of Wight, UK; 77 Regionie Puglia, Bari, Italy; 78 Faculdade de Medicina de Lisboa, Portuguese National Programme for Respiratory Diseases (PNDR), Lisbon, Portugal; 79 Geriatric Unit, Department of Internal Medicine (DIBIMIS), University of Palermo, Palermo, Italy; 80 Department of Medicine, University of Cape Town, Cape Town, South Africa; 81 Section of Respiratory Disease, Department of Oncology, Haematology and Respiratory Diseases, University of Modena and Reggio Emilia, Modena, Italy; 82 Department of Respiratory Medicine, Academic Medical Center (AMC), University of Amsterdam, Amsterdam, The Netherlands; 83 Department of Respiratory Medicine, National Institute of Diseases of the Chest and Hospital, Dhaka, Bangladesh; 84 Centre for Individualized Medicine, Department of Pediatrics, Faculty of Medicine, Linköping University, 58185 Linköping, Sweden; 85 Department of Dermatology and Allergy, Rheinische Friedrich-Wilhelms-University Bonn, Bonn, Germany; 86 Department of Respiratory Medicine and Allergology, University Hospital, Lund, Sweden; 87 Department of Geriatrics, Montpellier University Hospital, Montpellier, France; 88 EA 2991, Euromov, University Montpellier, Montpellier, France; 89 Department of Pathophysiology and Transplantation, IRCCS Fondazione Ca’Granda Ospedale Maggiore Policlinico, University of Milan, Milan, Italy; 90 Pediatric Department, University of Verona Hospital, Verona, Italy; 91 Department of Public Health and Infectious Diseases, Sapienza University of Rome, Rome, Italy; 92 Second University of Naples and Institute of Translational Medicine, Italian National Research Council, Naples, Italy; 93 La Rochelle, France; 94 Montreal, QC Canada; 95 Quebec Heart and Lung Institute, Laval University, Quebec City, QC Canada; 96 Health Economics and Health Technology Assessment, Institute of Health and Wellbeing, University of Glasgow, Glasgow, UK; 97 Institute of Lung Health, Respiratory Biomedical Unit, University Hospitals of Leicester NHS Trust, Leicestershire, UK; 98 Department of Infection, Immunity and Inflammation, University of Leicester, Leicester, UK; 99 Universitätsmedizin der Johannes Gutenberg-Universität Mainz, Mainz, Germany; 100 University of Medicine and Pharmacy Victor Babes, Timisoara, Romania; 101 Royal Brompton Hospital NHS, Imperial College London, London, UK; 102 Centro Medico Docente La Trinidad, Caracas, Venezuela; 103 National Heart and Lung Institute, Imperial College London, London, UK; 104 Montpellier, Région Languedoc Roussillon France; 105 S. Allergologia, S. Medicina Interna, Hospital Vall d’Hebron, Barcelona, Spain; 106 CEDOC, Respiratory Research Group, Nova Medical School, Campo dos Martires da Patria, Lisbon, Portugal; 107 Serviço de Imunoalergologia, Centro Hospitalar de Lisboa Central, EPE, Lisbon, Portugal; 108 Regional Ministry of Health of Andalusia, Seville, Spain; 109 Department of Paediatrics, Oslo University Hospital, Oslo, Norway; 110 University of Oslo, Oslo, Norway; 111 Allergy and Asthma Associates of Southern California, Mission Viejo, CA USA; 112 Allergy and Immunology Laboratory, Metropolitan University, Simon Bolivar University, Barranquilla, Colombia; 113 SLaai, Sociedad Latinoamericana de Allergia, Asma e Immunologia, Cartagena, Colombia; 114 Gérontopôle de Toulouse, 31059 Toulouse, France; 115 Department of Social Medicine, Faculty of Medicine, University of Crete, Heraklion, Crete Greece; 116 School of Medicine, University CEU San Pablo, Madrid, Spain; 117 Chachava Clinic, David Tvildiani Medical University-AIETI Medical School, Grigol Robakidze University, Tbilisi, Georgia; 118 Pulmonolory Research Institute FMBA, Moscow, Russia; 119 GARD Executive Committee, Moscow, Russia; 120 Medicine Department, IRCCS-Azienda Ospedaliera Universitaria San Martino, Genoa, Italy; 121 ICVS/3B’s-PT Government Associate Laboratory, Life and Health Sciences, Research Institute (ICVS), School of Health Sciences, University of Minho, Braga, Portugal; 122 Department of Medicine, Nova Southeastern University, Davie, FL USA; 123 EIP on AHA, European Innovation Partnership on Active and Healthy Ageing, Reference Site, Scottish Centre for Telehealth and Telecare, NHS 24, Glasgow, UK; 124 Department of Pediatric, Imperial College London, London, UK; 125 The Centre for Allergy Research, The Institute of Environmental Medicine, Karolinska Institutet, Stockholm, Sweden; 126 Department of Dermatology and Allergy, Technische Universität München, Munich, Germany; 127 ZAUM-Center for Allergy and Environment, Helmholtz Center Munich, Munich, Germany; 128 AQuAS, Barcelona, Spain; 129 EUREGHA, European Regional and Local Health Association, Brussels, Belgium; 130 Allergology and Immunology Discipline, “Luliu Hatieganu” University of Medicine and Pharmacy, Cluj-Napoca, Romania; 131 Division of Clinical Immunology and Allergy, Department of Medicine, McMaster University, Hamilton, ON Canada; 132 Peercode DV, Amsterdam, The Netherlands; 133 Respiratory Diseases Department, Rangueil-Larrey Hospital, Toulouse, France; 134 Service de Physiologie Respiratoire, Hôpital Cochin, Université Paris-Descartes, Assistance Publique-Hôpitaux de Paris, Paris, France; 135 University Clinic of Pulmology and Allergy, Medical Faculty, Ss Cyril and Methodius University, Skopje, Republic of Macedonia; 136 Service de Pneumo-Allergologie, Centre Hospitalo-Universitaire de Béni-Messous, Algers, Algeria; 137 Ecole des Mines, Alès, France; 138 Medical Faculty, Vilnius University, Vilnius, Lithuania; 139 Allergy and Clinical Immunology Section, National Heart and Lung Institute, Imperial College London, London, UK; 140 Guy’s and St Thomas’ NHS Trust, Kings College London, London, UK; 141 Section of Allergy and Immunology, Saint Louis University School of Medicine, Saint Louis, MO USA; 142 Computing Science Department, Umeå University, Umeå, Sweden; 143 Four Computing Oy, Halikko, Finland; 144 Pediatric Allergy and Immunology Unit, Ain Shams University, Cairo, Egypt; 145 Department of Health, Social Services and Public Safety, Belfast, Northern Ireland, UK; 146 Global Allergy and Asthma Platform GAAPP, Altgasse 8-10, 1130 Vienna, Austria; 147 Division of Allergy, Department of Pediatric Medicine, The Bambino Gesù Children’s Research Hospital Holy See, Rome, Italy; 148 Education for Health, Warwick, UK; 149 Department of Epidemiology, Regional Health Service Lazio Region, Rome, Italy; 150 Athens Chest Hospital, Athens, Greece; 151 National Center for Disease Control and Public Health of Georgia, Tbilisi, Georgia; 152 Department of Pulmonary Diseases, Cerrahpasa Faculty of Medicine, Istanbul University, Istanbul, Turkey; 153 Allergy and Immunology Division, Clinica Ricardo Palma, Lima, Peru; 154 Section of Allergology, Department of Internal Medicine, Erasmus MC, Rotterdam, The Netherlands; 155 Universidad Autónoma de Nuevo León, San Nicolás de los Garza, Mexico; 156 Center of Tuberculosis and Lung Diseases, Latvian Association of Allergists, Riga, Latvia; 157 Faculty of the Department of Neurology, University of Washington School of Medicine, Seattle, WA USA; 158 National eHealth Centre, University Hospital Olomouc, Olomouc, Czech Republic; 159 Immunology and Allergy Division Clinical Hospital, University of Chile, Santiago, Chile; 160 Department of Respiratory Diseases, Odense University Hospital, Odense, Denmark; 161 Institute of Epidemiology I, Helmholtz Zentrum München - German Research Center for Environmental Health, Neuherberg, Germany; 162 Vienna Challenge Chamber, Vienna, Austria; 163 Department of Paediatrics and Child Health, University College Cork, Cork, Ireland; 164 Université Paris-Sud, Le Kremlin Bicêtre, France; 165 Service de Pneumologie, Hôpital Bicêtre, Le Kremlin Bicêtre, France; 166 Inserm UMR_S999, Le Kremlin Bicêtre, France; 167 School of Psychology, Plymouth University, Plymouth, UK; 168 Department of Medicine and Surgery, University of Salerno, Baronissi, Italy; 169 Libra Foundation, Buenos Aires, Argentina; 170 Airway Disease Infection Section, National Heart and Lung Institute, Imperial College London, London, UK; 171 MRC & Asthma UK Centre in Allergic Mechanisms of Asthma, London, UK; 172 Department of Respiratory Medicine, Ghent University Hospital, Ghent, Belgium; 173 Medical Commission, Montpellier University Hospital, Montpellier, France; 174 Hallym University Sacred Heart Hospital, Hallym University College of Medicine, Anyang, Gyeonggi-do South Korea; 175 Department of Clinical Immunology, Wrocław Medical University, Wrocław, Poland; 176 Ukrainian Medical Stomatological Academy, Poltava, Ukraine; 177 Laboratory of Molecular Immunology, National Research Center, Institute of Immunology, Federal Medicobiological Agency, Moscow, Russia; 178 Pediatric Allergy and Asthma Unit, School of Medicine, Hacettepe University, Ankara, Turkey; 179 Immunology and Allergy Division, Department of Chest Diseases, School of Medicine, Hacettepe University, Ankara, Turkey; 180 First Department of Family Medicine, Medical University of Lodz, Lodz, Poland; 181 Department of Medicine, McMaster University, Health Sciences Centre 3V47, 1280 Main Street West, Hamilton, ON Canada; 182 Department of Pulmonary Diseases, University Medical Center Groningen, University of Groningen, Groningen, The Netherlands; 183 GARD, Geneva, Switzerland; 184 Department of Respiratory Medicine, Faculty of Medicine and Dentistry, University Hospital Olomouc, Olomouc, Czech Republic; 185 Department of Pediatric Pulmonology and Pediatric Allergology, Beatrix Children’s Hospital, GRIAC Research Institute, University Medical Center Groningen, University of Groningen, Groningen, The Netherlands; 186 Department of Immunology, Rheumatology and Allergy and HARC, Medical University of Lodz, Lodz, Poland; 187 Children’s Hospital, University of Helsinki, Helsinki, Finland; 188 Clinic of Infectious, Chest Diseases, Dermatology and Allergology, Vilnius University, Vilnius, Lithuania; 189 VIB Inflammation Research Center, Ghent University, Ghent, Belgium; 190 Department for Pediatric Pneumology and Immunology, Charité Medical University, Berlin, Germany; 191 University of Medicine and Pharmacy, Hochiminh City, Vietnam; 192 Divisions of Allergy and Immunology, Department of Internal Medicine and Pediatrics, University of Tennessee College of Medicine, Germantown, TN USA; 193 Scottish Centre for Respiratory Research, Cardiovascular and Diabetes Medicine, Medical Research Institute, Ninewells Hospital, University of Dundee, Dundee, UK; 194 State Key Laboratory of Respiratory Diseases, Guangzhou Institute of Respiratory Disease, The First Affiliated Hospital of Guangzhou Medical University, Guangzhou, China; 195 Institute of Clinical Medicine, Faculty of Medicine, University of Oslo, Oslo, Norway; 196 Department of Pulmonary Medicine, CHU Sart-Tilman, Liege, Belgium; 197 Division of Immunopathology, Department of Pathophysiology and Allergy Research, Center for Pathophysiology, Infectiology and Immunology, Medical University of Vienna, Vienna, Austria; 198 The Queen’s Medical Research Institute, University of Edinburgh, Edinburgh, UK; 199 Service de Pneumo-allergologie, Hôpital Saint-Joseph, Paris, France; 200 Service de Pneumologie, UMR INSERM, UMR1087 and CNR 6291, l’institut du Thorax, University of Nantes, Nantes, France; 201 Department of Pulmonary Medicine, Rashid Hospital, Dubai, UAE; 202 Biomax Informatics AG, Munich, Germany; 203 Department of Respiratory Medicine, University of Bratislava, Bratislava, Slovakia; 204 Institute of Biomedical Imaging and Life Sciences (IBILI), Faculty of Medicine, University of Coimbra, Coimbra, Portugal; 205 Ageing@Coimbra EIP-AHA Reference Site, Coimbra, Portugal; 206 Department of Medicine (RCSI), Bon Secours Hospital, Glasnevin, Dublin, Ireland; 207 Kronikgune, Basque Region, Spain; 208 Laboratory of Behavioral Immunology Research, Division of Clinical Immunology and Allergy, The University of Mississippi Medical Center, Jackson, MS USA; 209 Tobacco Control Research Centre, Iranian Anti Tobacco Association, Tehran, Iran; 210 Department of Paediatrics, Maputo Central Hospital, Maputo, Mozambique; 211 Allergy and Asthma Medical Group and Research Center, San Diego, CA USA; 212 Department of Physiology, CHRU, PhyMedExp, INSERM U1046, CNRS UMR 9214, University Montpellier, Montpellier, France; 213 Hautklinik - Klinik für Dermatologie & Allergologie, Universitätsklinikum der RWTH Aachen, Aachen, Germany; 214 Croatian Pulmonary Society, Zagreb, Croatia; 215 National Institute of Pneumology M. Nasta, Bucharest, Romania; 216 Faculty of Medicine, University of Belgrade, Belgrade, Serbia; 217 Serbian Association for Asthma and COPD, Belgrade, Serbia; 218 Caisse d’assurance retraite et de la santé au travail du Languedoc-Roussillon (CARSAT-LR), Montpellier, France; 219 National Center for Research in Chronic Respiratory Diseases, Tishreen University School of Medicine, Latakia, Syria; 220 Department of Public Health and Health Products, EA 4064, Paris Descartes University-Sorbonne Paris Cité, Paris, France; 221 Paris Municipal Department of Social Action, Childhood, and Health, Paris, France; 222 Institute of Medical Statistics, Informatics and Epidemiology, Medical Faculty, University of Cologne, Cologne, Germany; 223 Food Allergy Referral Centre Veneto Region, Department of Women and Child Health, Padua General University Hospital, Padua, Italy; 224 Scientific Centre of Children’s Health Under the Russian Academy of Medical Sciences, Moscow, Russia; 225 Hospital de Niños Orlando Alassia, Santa Fe, Argentina; 226 Hospital of the Hospitaller Brothers in Buda, Budapest, Hungary; 227 Neumología y Alergología Infantil, Hospital La Fe, Valencia, Spain; 228 Department of Internal Medicine, Centro Hospitalar Sao Joao, Porto, Portugal; 229 ENT Department, University Hospital of Kinshasa, Kinshasa, Congo; 230 National Hospital Organization, Tokyo National Hospital, Tokyo, Japan; 231 Department of Otorhinolaryngology, Chiba University Hospital, Chiba, Japan; 232 Department of Otolaryngology, Nippon Medical School, Tokyo, Japan; 233 Centre Hospitalier Universitaire Pédiatrique Charles de Gaulle, Ouagadougou, Burkina Faso; 234 Cardio-Thoracic and Vascular Department, University Hospital of Pisa, Pisa, Italy; 235 Department of Comparative Medicine, Messerli Research Institute of the University of Veterinary Medicine, Medical University, Vienna, Austria; 236 Department of Immunology and Allergology, Faculty of Medicine and Faculty Hospital in Pilsen, Charles University in Prague, Pilsen, Czech Republic; 237 Respiratory Medicine, Department of Medical Sciences, University of Ferrara, Ferrara, Italy; 238 Department of Allergy and Clinical Immunology, Ajou University School of Medicine, Suwon, South Korea; 239 University of Southern Denmark, Kolding, Denmark; 240 Allergy Unit, CUF-Porto Hospital and Institute, Porto, Portugal; 241 Department of Otorhinolaryngology, Head and Neck Surgery, Universitätsmedizin Mannheim, Medical Faculty Mannheim, Heidelberg University, Mannheim, Germany; 242 Conseil Général de l’Economie, Ministère de l’Economie, de l’Industrie et du Numérique, Paris, France; 243 Société de Pneumologie de Langue Française, Espace francophone de Pneumologie, Paris, France; 244 Département de pédiatrie, CHU de Grenoble, Grenoble, France; 245 Children’s Hospital Srebrnjak, Zagreb, Croatia; 246 School of Medicine, University J.J. Strossmayer, Osijek, Croatia; 247 Karl Landsteiner Institute for Clinical and Experimental Pneumology, Hietzing Hospital, Vienna, Austria; 248 Clinic of Allergy and Asthma, Medical University Sofia, Sofia, Bulgaria; 249 University Medical Center Groningen, University of Groningen, Groningen, The Netherlands; 250 Laboratory of Medical Allergology, Allergy Clinic, Copenhagen University Hospital at Gentofte, Copenhagen, Denmark; 251 Academic Centre of Primary Care, University of Aberdeen, Aberdeen, Scotland, UK; 252 Research in Real-Life, Cambridge, UK; 253 LungenClinic Grosshansdorf, Airway Research Center North, German Center for Lung Research (DZL), Grosshansdorf, Germany; 254 Department of Medicine, Christian Albrechts University, Airway Research Center North, German Center for Lung Research (DZL), Kiel, Germany; 255 NHS Foundation Trust, University Hospitals of Southampton, Southampton, UK; 256 Centre of Pneumology, Coimbra University Hospital, Coimbra, Portugal; 257 Polibienestar Research Institute, University of Valencia, Valencia, Spain; 258 Department of Geriatrics, Getafe University Hospital, Madrid, Spain; 259 Association Asthme et Allergie, Paris, France; 260 Primary Care Respiratory Research Unit, Institutode Investigación Sanitaria de Palma IdisPa, Palma de Mallorca, Spain; 261 Allergy Unit, Complesso Integrato Columbus, Rome, Italy; 262 Serviço de Imunoalergologia, Hospital da Luz, Lisbon, Portugal; 263 Hospital de Clinicas, University of Parana, Curitiba, Brazil; 264 Division of Allergy Asthma and Clinical Immunology, Emek Medical Center, Afula, Israel; 265 Allergy and Clinical Immunology Department, Centro Médico-Docente La Trinidad and Clínica El Avila, Caracas, Venezuela; 266 Faculty of Medicine, Autononous University of Madrid, Madrid, Spain; 267 The Royal National TNE Hospital, University College London, London, UK; 268 DIBIMIS, University of Palermo, Palermo, Italy; 269 Allergy Unit, Department of Dermatology, University Hospital of Zurich, Zurich, Switzerland; 270 Otolaryngology and Head and Neck Surgery, CHU Rangueil-Larrey, Toulouse, France; 271 Child Health, Queen’s University, Belfast, Northern Ireland, UK; 272 Royal Belfast Hospital for Sick Children, Belfast, Northern Ireland, UK; 273 INSERM, Université Grenoble Alpes, IAB, U 1209, Team of Environmental Epidemiology Applied to Reproduction and Respiratory Health, Université Joseph Fourier, Grenoble, France; 274 Sociedad Paraguaya de Alergia Asma e Inmunologıa, Asunción, Paraguay; 275 Julius Center of Health Sciences and Primary Care, University Medical Center Utrecht, University of Utrecht, Utrecht, The Netherlands; 276 Division of Allergy, Clinical Immunology and Rheumatology, Department of Pediatrics, Federal University of São Paulo, São Paulo, Brazil; 277 Kyrgyzstan National Centre of Cardiology and Internal Medicine, Euro-Asian Respiratory Society, Bishkek, Kyrgyzstan; 278 Pulmonary Division, Heart Institute (InCor), Hospital da Clinicas da Faculdade de Medicina da Universidade de Sao Paulo, São Paulo, Brazil; 279 Academic Medical Centre, University of Amsterdam, Amsterdam, The Netherlands; 280 European Union Geriatric Medicine Society (EUGMS), Helsinki, Finland; 281 Department of Epidemiology, CAPHRI School of Public Health and Primary Care, Maastricht University, Maastricht, The Netherlands; 282 Pneumology and Allergy Department, Hospital Clínic, Clinical and Experimental Respiratory Immunoallergy, IDIBAPS, Barcelona, Spain; 283 PELyon, Lyon, France; 284 HESPER 7425, Health Services and Performance Resarch, Université Claude Bernard Lyon, Villeurbanne, France; 285 University Hospital, Stockholm, Sweden; 286 Department of Chest Medicine, Centre Hospitalier Universitaire UCL Namur, Université Catholique de Louvain, Yvoir, Belgium; 287 Philippines Society of Allergy, Asthma and Immunology, Manila, Philippines; 288 Pulmonary Unit, Department of Cardiology, Thoracic and Vascular Medicine, Arcispedale S. Maria Nuova/IRCCS, Research Hospital, Reggio Emilia, Italy; 289 Regional Agency for Health and Social Care, Reggio Emilia, Italy; 290 Finnish Lung Association (FILHA), Helsinki, Finland; 291 Pulmonary Environmental Epidemiology Unit, CNR Institute of Clinical Physiology, Pisa, Italy; 292 CNR Institute of Biomedicine and Molecular Immunology “A. Monroy”, Palermo, Italy; 293 Sotiria Hospital, Athens, Greece; 294 Department of Otorhinolaryngology, HNO-Klinik, Universitätsklinikum Düsseldorf, Düsseldorf, Germany; 295 Asthma UK, Mansell Street, London, UK; 296 Department of Otolaryngology, Yong Loo Lin School of Medicine, National University of Singapore, Singapore, Singapore; 297 Division of Immunodermatology and Allergy Research, Department of Dermatology and Allergy, Hannover Medical School, Hannover, Germany; 298 Eshelman School of Pharmacy, University of North Carolina, Chapel Hill, NC USA; 299 IPCRG, Aberdeen, Scotland, UK; 300 Bradford Institute for Health Research, Bradford Royal Infirmary, Bradford, UK; 301 Department of Research, Olmsted Medical Center, Rochester, MN USA; 302 Medical School, University of Cyprus, Nicosia, Cyprus; 303 The Allergy and Asthma Institute, Lahore, Pakistan; 304 Social Sciences, University of Southampton, Southampton, UK; 305 Department of Paediatrics and Child Health, Red Cross Children’s Hospital, University of Cape Town, Cape Town, South Africa; 306 MRC Unit on Child and Adolescent Health, University of Cape Town, Cape Town, South Africa; 307 Universidad Católica de Córdoba, Córdoba, Argentina; 308 Department of Otolaryngology Head and Neck Surgery, Beijing TongRen Hospital, Beijing, China; 309 Beijing Institute of Otolaryngology, Beijing, China; 310 University Clinic of Respiratory and Allergic Diseases, Golnik, Slovenia; 311 Northern Health Alliance, Newcastle, UK; 312 CHRU Arnaud de Villeneuve, 371 Avenue du Doyen Gaston Giraud, 34295 Montpellier Cedex 5, France

**Keywords:** ARIA, Rhinitis, ICT, EIP on AHA, Mobile technology, AIRWAYS ICPs

## Abstract

The Allergic Rhinitis and its Impact on Asthma (ARIA) initiative commenced during a World Health Organization workshop in 1999. The initial goals were (1) to propose a new allergic rhinitis classification, (2) to promote the concept of multi-morbidity in asthma and rhinitis and (3) to develop guidelines with all stakeholders that could be used globally for all countries and populations. ARIA—disseminated and implemented in over 70 countries globally—is now focusing on the implementation of emerging technologies for individualized and predictive medicine. MASK [MACVIA (*Contre les Maladies Chroniques pour un Vieillissement Actif*)-ARIA Sentinel NetworK] uses mobile technology to develop care pathways for the management of rhinitis and asthma by a multi-disciplinary group and by patients themselves. An app (Android and iOS) is available in 20 countries and 15 languages. It uses a visual analogue scale to assess symptom control and work productivity as well as a clinical decision support system. It is associated with an inter-operable tablet for physicians and other health care professionals. The scaling up strategy uses the recommendations of the European Innovation Partnership on Active and Healthy Ageing. The aim of the novel ARIA approach is to provide an active and healthy life to rhinitis sufferers, whatever their age, sex or socio-economic status, in order to reduce health and social inequalities incurred by the disease.

## Background

The Allergic Rhinitis and its Impact on Asthma (ARIA) initiative commenced during a World Health Organization (WHO) workshop in 1999 (published in 2001) [1]. The goals were (1) to propose a new allergic rhinitis (AR) classification using persistence and severity of symptoms in order to more closely reflect patients’ needs, (2) to promote the concept of multi-morbidity in asthma and rhinitis as a key factor for patient management, (3) to develop guidelines with all stakeholders, (4) to include experts from developed and developing countries and (5) to initiate global implementation among health care professionals (HCPs) and patients.

Patients, clinicians and other HCPs are confronted with various treatment choices for the management of AR. This contributes to considerable variation in clinical practice. Worldwide, patients, clinicians and other HCPs are faced with uncertainty about the relative merits and downsides of the many AR treatment options available. The first ARIA workshop report used the Shekelle evidence-based methodology [1, 2]. It was the first guideline in chronic disease to assess multi-morbid conditions (i.e. asthma and rhinitis in the same patient). In 2008, ARIA was updated using the same evidence-based system [3]. More transparent reporting of guidelines to facilitate understanding and acceptance was needed. In its 2010 Revision, ARIA was the first chronic respiratory disease guideline to adopt the GRADE (Grading of Recommendation, Assessment, Development and Evaluation) approach, an advanced evidence evaluation methodology [4–7]. A new revision is pending.

ARIA has been disseminated and is implemented in over 70 countries around the world [8]. It is now focusing on the implementation of emerging technologies for individualized and predictive medicine. MASK [MACVIA (*Contre les Maladies Chroniques pour un Vieillissement Actif*)-ARIA Sentinel NetworK] uses mobile technology to develop care pathways for the management of rhinitis and asthma by a multi-disciplinary group and by patients themselves [9].

The aim of the novel ARIA approach is to provide an active and healthy life to rhinitis sufferers, whatever their age, sex or socio-economic status in order to reduce health and social inequalities incurred by the disease.

## AIRWAYS ICPs: the ARIA 2016 political agenda

In 2012, the European Commission launched the European Innovation Partnership on Active and Healthy Ageing (EIP on AHA; DG Santé and DG CONNECT) to enhance EU competitiveness and tackle societal challenges through research and innovation [10]. The B3 Action Plan is devoted to the scaling up and replication of successful innovative integrated care models for chronic diseases amongst older patients.

Chronic respiratory diseases were selected to be the pilot for chronic diseases of the EIP on AHA Action Plan B3 (Integrated care pathways for airway diseases, AIRWAYS ICPs) [11, 12] with a life cycle approach [13]. Several effective plans exist in Europe for chronic respiratory diseases, but they are rarely deployed to other regions or countries.

AIRWAYS ICPs aims to launch a collaboration to develop practical multi-sectoral care pathways (i.e. ICPs) in European countries and regions to reduce chronic respiratory disease burden, mortality and multi-morbidity, while maintaining patients’ quality-of-life (QOL) [11, 14]. AIRWAYS-ICPs proposes a feasible, achievable and manageable project (from science to guidelines and policies) using existing networks. It brings together key stakeholders including end users, public authorities, industry partners, involved in the innovation cycle, from research to adoption, as well as those engaged in standardisation and regulation. The Action Plan of AIRWAYS ICPs has been devised [11], implemented [14] and scaled up [15].

AIRWAYS ICPs is a GARD (WHO Global Alliance against Chronic Respiratory Diseases) [16] research demonstration project. Its deployment beyond Europe is carried out via GARD.

One AIRWAYS-ICPs activity is the development of multi-sectoral care pathways for rhinitis and asthma and their multi-morbidities, implementing emerging technologies for predictive medicine across the patient life cycle [13].

## From guidelines to integrated care pathways: MACVIA-ARIA Sentinel NetworK (MASK)

### Best practice, guideline and care pathways

A *good* or *best practice* is a technique, method, process, activity, incentive, or reward believed to be more effective than any other technique, method, process, etc. when applied to a particular condition or circumstance. A best practice can be adopted as a standard process or be used as a guideline (U.S. Dept. of Veterans Affairs [17]).

A *guideline* is a statement to determine a course of action. It aims to streamline particular processes according to a set routine or sound practice. By definition, following a guideline is never mandatory. Guidelines are not binding and are not enforced (U.S. Dept. of Veterans Affairs [17]).

“*Clinical practice guidelines* are systematically developed statements to assist practitioner and patient decisions about appropriate health care for specific clinical circumstances” (Institute of Medicine, 1990). These clinical practice guidelines define the role of specific diagnostic and treatment modalities. The statements include recommendations based on evidence intended to help HCPs and providers in their practice [18].

*The Integrated Care Pathway* (ICP) concept was initiated in 1985 by Zander and Bower [19]. ICPs are structured multi-disciplinary care plans detailing key steps of patient care for a given clinical problem [20]. They promote the translation of guidelines into local protocols and their subsequent application to clinical practice. An ICP forms all or part of the clinical record, documents the care given, and facilitates the evaluation of outcomes for continuous quality improvement [21]. They can help empower patients and their carers (health and social). ICPs differ from clinical practice guidelines as they are utilized by a multi-disciplinary team, and focus on the quality and co-ordination of care. ICPs need to have a mechanism for recording variations/deviations from planned care. Like guidelines, an ICP is a guide to treatment, and clinicians are free to exercise their own professional judgment as appropriate. However, any alteration to the practice identified within this ICP must be noted as a variance [22]. Variance analysis is a critical part of developing and using ICPs [23]. The resulting analysis can be used to amend the ICP itself if, for the majority of patients, the practice is different to the pathway (Table 1).

**Table 1:** Definition of guidelines, practice protocols and ICPs. Adapted from http://www.implementationcentral.com/guidelines_8.html

	Guideline	Clinical practice guidelines	Care pathway
Focus	Specific clinical circumstances	Treatment and/or prevention	The quality and co-ordination of care
Definition	Systematically developed statements to help practitioners and patients make decisions about appropriate health care	A suggested course of treatment and/or treatment service for a specific diagnosis, functional deficit or problem area	Structured, multi-disciplinary plans of care
Goals	Makes specific recommendations on health care and links these to research evidence	Highlights major therapeutic or preventive interventions Identifies choices of different courses or paths of treatment	Supports the implementation of clinical guidelines and protocols
Outputs	Provides a summary and appraisal of the best available research evidence or expert consensus Highlights the strength of the evidence underlying each recommendation Describes barriers and facilitators for each recommendation	Provides a logical flow of interventions. Provides detailed recommendations that build on those made in SPCs guidelines	Provides detailed guidance for each stage in the management of a patient and key performance indicators
Users	Clinicians, patients and third parties (all stakeholders involved)	Specific to clinicians	A multidisciplinary clinical team
Components	(1) Appraisal of literature (research evidence or expert consensus) (2) Summary of recommendations (3) An outline of how guidelines should be implemented and how adherence monitored	List of major therapeutic or preventive interventions Goals: When interventions should be achieved Options for different choices of treatment and/or prevention	(1) Timeline (2) Categories of care/intervention (3) Intermediate and long term outcome criteria (4) A variance record

### Multisectoral care pathways for rhinitis and asthma using ICT

**Fig. 1:**
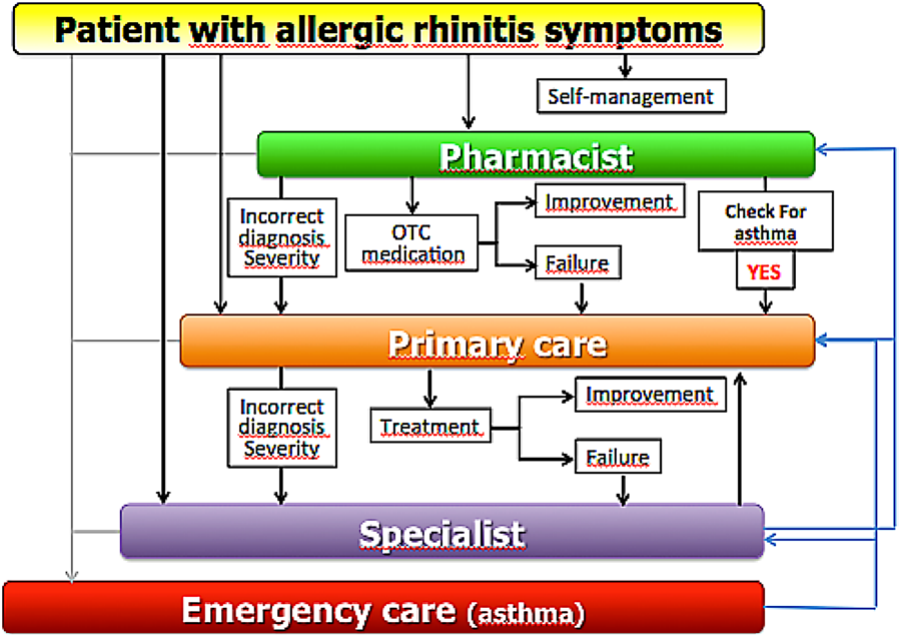
Multi-sectoral care pathway for allergic rhinitis (from Bousquet et al. [9])

A large number of AR patients do not consult physicians because they think their AR symptoms are ‘normal’ and/or trivial. However, AR negatively impacts social life, school and work productivity [3]. Many AR patients use over the counter (OTC) drugs [24] and only a fraction have had a medical consultation. The vast majority of patients who visit GPs or specialists have moderate/severe rhinitis [25–29]. Thus, ICPs should consider a multi-disciplinary approach as proposed by AIRWAYS ICPs (Fig. 1).

The variations/deviations of the ARIA recommendations from planned care have been assessed and several unmet needs identified. Disease severity is associated with several health outcomes, including quality of life [25–29], and should be considered in ICPs. The duration of rhinitis (intermittent/severe-persistent) is an important indicator of asthma multi-morbidity (in some but not all studies) [30], duration of AR treatment and efficacy of treatment in AR [27]. Most patients receive combinations of oral antihistamines and intra-nasal corticosteroids (INS) [31–33] which are not evaluated in guidelines using an appropriate methodology.

### Simple approach to assess control in allergic rhinitis

In asthma, the management strategy is based on disease control, current treatment and future risk (exacerbations, lung function decline) [34–36]. In AR, the switch from symptom severity to disease control to guide treatment decisions has been led by ARIA and is now expanding to include MACVIA (a reference site of the EIP on AHA, EU [37]) to form an Action Plan (MACVIA-ARIA). New developments which have facilitated this process include (1) the introduction of the visual analogue scale (VAS) as the common validated language of AR control, (2) categorization of AR control using VAS score cut-offs, (3) incorporation of this VAS into simple interactive apps for both patients (*ARIA Allergy Diary*) and HCPs (*ARIA Allergy Diary Companion*) [9, 38] and (4) the integration of all this knowledge into ICPs deployed by the EIP on AHA [9].

The VAS represents a simple way of measuring control. It has been used in many diseases, including AR. VAS scores appear to be similar in different countries, for patients with moderate–severe intermittent or persistent rhinitis [39, 40]. An advantage of the VAS is that it can be used in all age groups, including preschool children (guardian evaluation) [41] and the elderly [42, 43]. Furthermore, it can be used in a wide variety of languages [39, 40, 42, 44–48]. VAS scores vary with ARIA AR classification in many languages [28, 44, 49, 50]. A VAS score of 50/100 mm suggests moderate–severe AR [32, 51, 52], although in some studies this cut-off was >60 mm [45]. The VAS has been used to define severe chronic upper airway disease (SCUAD [53]). The minimal clinically important difference (MCID) during treatment was found to be 2.3/10 cm in the French population [54] and may be generalized to other countries, but future studies may refine this cut-off score. VAS score changes appear to encompass both symptoms and disease-specific QOL [54, 55].

As is the case for asthma, the best control of AR should be achieved as early as possible in order to (1) improve patient satisfaction and concordance with treatment and (2) reduce the AR burden including symptoms, reduced QoL, and school and work presenteeism/absenteeism. Untreated AR can impair driving ability and put patients at risk [56]. The ultimate goal of AR control is to reduce the direct and indirect costs incurred by AR [57–60].

The variability in approaches to achieve disease control is challenging, and necessitates careful monitoring as well as the step up/step down of individualized therapeutic regimens over time. However, the challenges of managing AR are increased by the fact that patients do not often recognise their AR symptoms or confuse them with those of asthma or other multimorbidities such as rhinosinusitis [61]. Therefore, it is important for patients, caregivers or HCPs to be able to use an AR symptom scoring system that is simple to use and rapidly responsive to change.

The aim is to encourage effective cross communication and achieve rapid and sustained disease control. MACVIA-ARIA has produced a simple VAS-based algorithm called the *ARIA Clinical Decision Support System* (CDSS) using a VAS score to guide treatment decisions in a step-up/step-down approach. This CDSS provides an individualized approach to AR pharmacotherapy (depending on medication availability and resources) [62]. This approach holds the potential for optimal AR control while minimizing side effects and costs.

### MASK (MACVIA-ARIA Sentinel NetworK): rhinitis and asthma

MASK-rhinitis and asthma is a simple ICT tool used to implement ICPs for AR and asthma by means of a common language (for patients and HCPs) and a CDSS. Disease control is assessed by VAS, incorporated into apps for patients (*ARIA Allergy Diary*) and HCPs (*ARIA Allergy Diary Companion*), with the utility to assess patient QoL (weekly EQ-5D) [63, 64] and school/work productivity (weekly WPAI-AS and daily VAS) [25, 65, 66].

MASK-rhinitis and asthma will (1) allow patients and caregivers to screen for AR and asthma, and track their AR control (2) guide pharmacists in the prescription of OTC medications and referral of patients to physicians when appropriate, (3) allow primary care physicians to prescribe appropriate AR treatment, assess patients’ AR control and direct follow-ups in accordance with the CDSS and (4) encourage referral to specialists and outpatient clinics, if there is failure to gain AR control at the primary care level.

MASK-rhinitis and asthma will be important for establishing care pathways across the life cycle. It will stratify patients with severe uncontrolled disease and achieve better results in prevention and intervention trials guided by the use of an individualised and predictive medicine approach.

### The MASK tools: the *ARIA Allergy Diary* and *ARIA Allergy Diary Companion* apps

*The ARIA Allergy Diary* is freely available in 15 EU countries, Australia, Mexico and Switzerland and in 15 languages (translated and back-translated, culturally adapted and legally compliant). It will also be deployed in Brazil, Canada and the USA. The companion app will be available in Autumn 2016.

A pilot study was completed in AR during the pollen season to assess the relevance of the *ARIA Allergy Diary* app. It showed the importance of the tool to stratify patients, assess their work productivity and improve quality of life (EQ-5D) (Bousquet et al., submitted). Studies in asthma are planned for the autumn and winter.

#### Questionnaires

*ARIA Allergy Diary* users fill in simple questionnaires on asthma, rhinitis and the impact of the disease (globally, on work and school, on daily activities and on sleep) upon registration (Table 2). The pilot study in around 5000 users (9% over 60 years of age) indicates that these questions are easily answered and can help to stratify patients with rhinitis.

**Table 2:** Baseline questionnaire

Q1: I have rhinitis: yes/no
Q2: I have asthma: yes/no
Q3: My symptoms (tick)
Runny nose
Itchy nose
Sneezing
Congestion (blocked nose)
Red eyes
Itchy eyes
Watery eyes
Q4: How they affect me: my symptoms (tick)
Affect my sleep
Restrict my daily activities
Restrict my participation in school or work
Are troublesome
Q5: Medications
Q6: Are you currently receiving immunotherapy (a small dose of the thing you are allergic to, usually taken as an injection or placed under your tongue)? yes/no
If YES to Q6 (Q7 and Q8)
Q7: What allergy is this?
Grass pollen
Parietaria pollen
Birch pollen
Other pollen
Dust mite
Animal
Cypress tree pollen
Don’t know
Add allergy
Q8: How do you receive your treatment?
Injection
Tablet under the tongue
Drops under the tongue
Spray under the tongue
Other

Moreover, two specific questionnaires are applied every week to assess disease impact on patients’ QoL (EQ-5D) [63, 64] and productivity at work (WPAI-AS) [25, 65, 66].

#### Treatments received

**Fig. 2:**
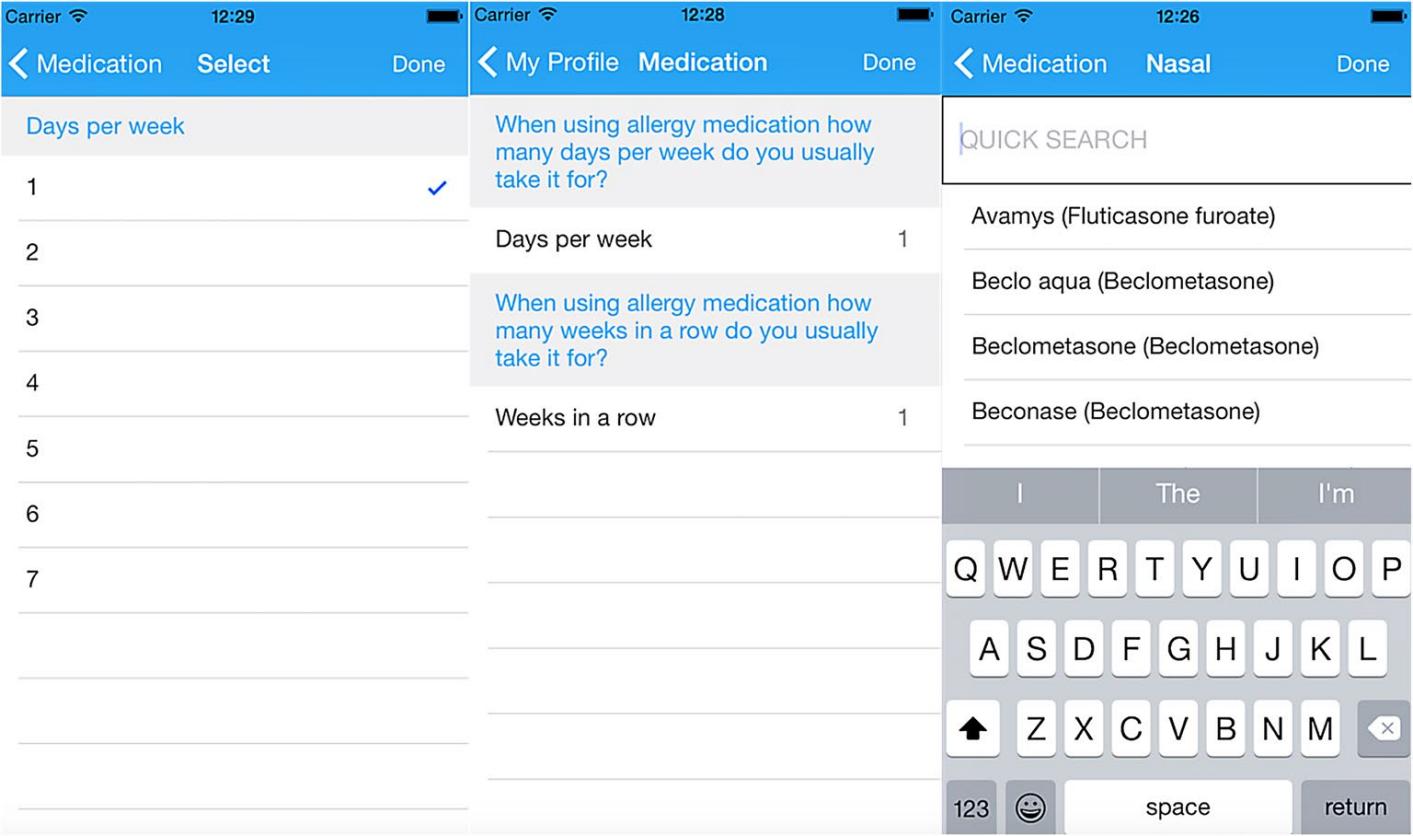
Screens on medications

A list of all treatments available for asthma, conjunctivitis and rhinitis is included in the *ARIA Allergy Diary*, and users select the treatment(s) they are taking. Multiple treatments may be selected, and users can update the information when (or if) their treatment changes (Fig. 2). The list has been customized for all 20 countries in which the *ARIA Allergy Diary* is available, using data from IMS Health. Information on allergen specific immunotherapy is also requested on the day of first use. A questionnaire on biologics for asthma is under development.

#### Daily visual analogue scales

**Fig. 3:**
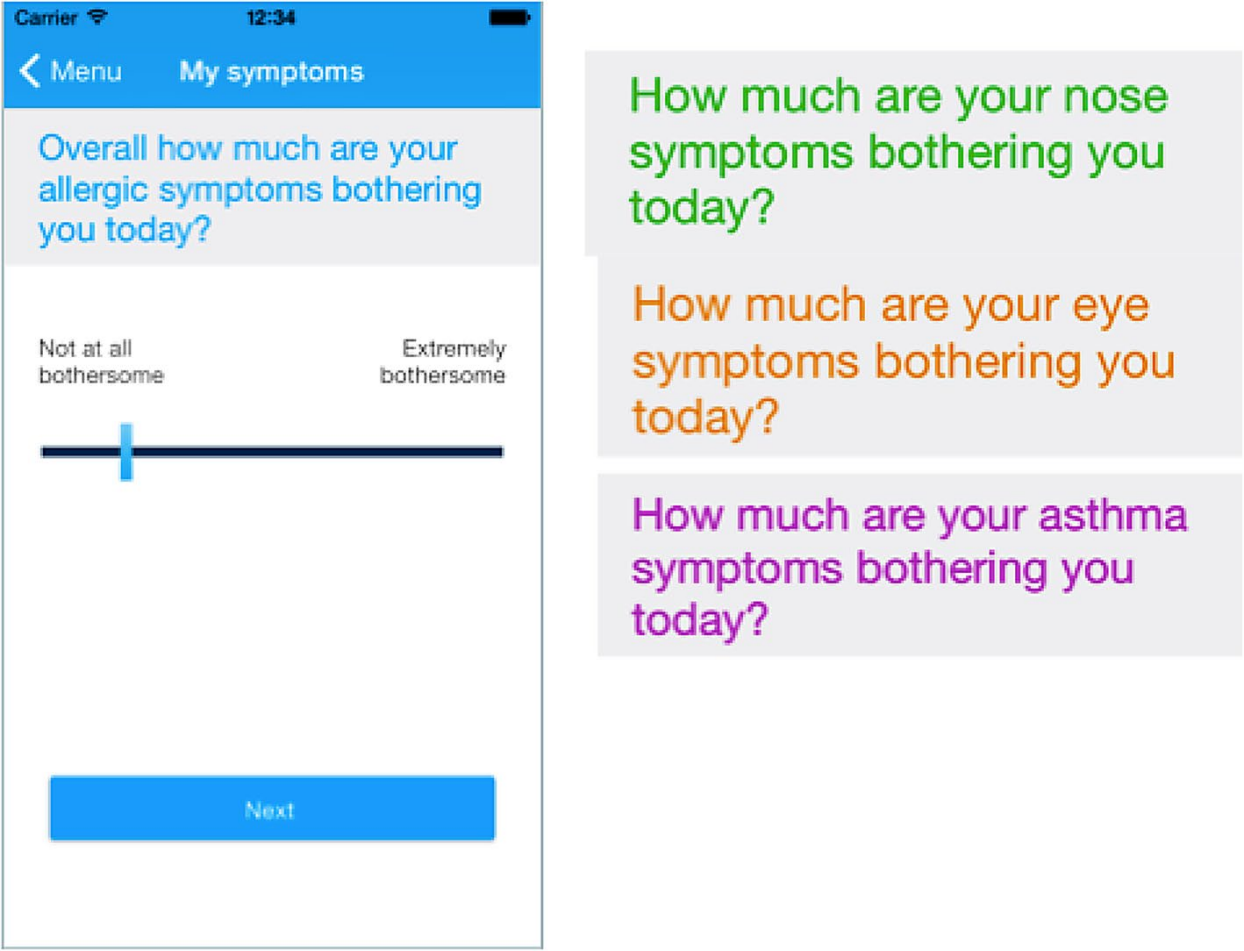
Screens on daily visual analogue scales

Geolocalized users assess their daily symptom control using the touchscreen functionality on their smart phone to click on 5 consecutive VASs (global symptoms due to allergic diseases, rhinitis, conjunctivitis, asthma and work productivity) (Fig. 3). These scales have been validated for AR and asthma criteria [67–71] and for work productivity (Bousquet et al., in preparation).

#### MASK-asthma

Besides the asthma VAS, a test to measure pulmonary function is being developed. It is expected to be added to the *ARIA Allergy Diary* by the end of 2016.

#### Clinical decision support system

The MASK CDSS is incorporated into an app for HCPs (i.e. *ARIA Allergy Diary Companion*). This is essentially an algorithm to aid clinicians to select pharmacotherapy for patients with AR and to stratify their disease severity [62]. It uses a simple step-up/step-down individualized approach to AR pharmacotherapy and may hold the potential for optimal control of symptoms, while minimizing side effects and costs. However, its use varies depending on the availability of medications in the different countries and on resources. A CDSS for asthma is also being developed.

#### Ethics

The terms of use have been translated into all languages and customized according to the country’s legislation. They allow the use of anonymous data for research and commercial purposes. The app has a CE registration as a medical device class 1.

#### Patient empowerment

The validation of the *ARIA Allergy Diary* has already been accomplished (manuscript in preparation). With the help of patient organisations (EFA: European Federation of Allergy and Airways Diseases Patients’ Associations), it will be evaluated and improved by the patients themselves.

## New concepts in allergic multimorbidity embedded in ARIA

The term allergic multimorbidity is more appropriate than comorbidity since the primary allergic disease is poorly known [72].

### Stratification of severe allergic and/or asthma patients

Despite the major advances in understanding allergic diseases or asthma, treatments are not effective in all patients. From a clinical perspective, implementing knowledge-based decisions on what therapeutics to use for which patients and, if relevant, in which combinations, is extremely challenging. The aspiration to provide more effective therapeutic interventions tailored to the individual remains unfulfilled because of the variable response of individuals to such interventions. Patient stratification aims at grouping patients into disease sub-groups, where the specific pathological processes involved are better defined (clinical/molecular phenotypes).

Long-term birth cohort studies are essential for understanding the life course, early predictors, risk and protective factors of allergic diseases (including asthma and rhinitis) and the complex interplay between genes and environment (including life style and socio-economic determinants) [73]. MeDALL (Mechanisms of the Development of ALLergy; EU FP7-CP-IP; Project No.: 261357; 2010–2015) attempted to better understand the complex links of allergic diseases at the clinical and mechanistic levels [74–76].

MeDALL identified a rare but severe allergy phenotype: polysensitized-multimorbid phenotype. Although multimorbidity is not always associated with allergy, studies in MeDALL [77] on children, in the PARIS cohort at 2 years of age [78], in EGEA on adults [79, 80] (Siroux, in preparation) and patient cohorts in subjects with peanut allergy [81] all show that subjects who are polysensitized and multimorbid have a very high frequency of allergic symptoms, persistent symptoms over time, more severe asthma symptoms than other phenotypes and higher total and specific IgE levels.

Taken altogether, these results indicate that asthmatic patients cannot be managed appropriately without assessing rhinitis multimorbidity and also reinforce the importance of nasal problems (rhinitis and/or rhinosinusitis) in many uncontrolled asthmatic patients [82–84].

### Allergic multimorbidity in old age adults

Asthma and rhinitis often start in early age and persist in most, but not all, subjects. The expected epidemic wave of asthma and rhinitis in older adults is an insufficiently recognized problem. In Europe, over 20% of adults suffer from AR and over 5% from asthma. These patients are now reaching the age of 65 years and a new health problem in older adults will be to understand, detect and manage these patients. Asthma and rhinitis in older adults have specific symptoms and treatment needs, which are different from those in younger adults. These patients also suffer from multi-morbididy with high rates of poly-pharmacy reported. Integrated Care Pathways (ICPs) for rhinitis and asthma should cover the entire life cycle.

## The scaling up strategy

The EIP on AHA has proposed a 5-step framework for developing an individual scaling up strategy: (1) what to scale up: (1-a) databases of good practices, (1-b) assessment of viability of the scaling up of good practices, (1-c) classification of good practices for local replication and (2) how to scale up: (2-a) facilitating partnerships for scaling up, (2-b) implementation of key success factors and lessons learnt, including emerging technologies for individualised and predictive medicine. This strategy has already been applied to the chronic respiratory diseases action plan of the EIP on AHA [15].

There is an urgent need for scaling up strategies in order to (1) avoid fragmentation, (2) improve health care delivery across Europe, (3) speed up the implementation of good practices using existing cost-effective success stories and (4) meet the EIP on AHA objectives [10].

### Reference Site Collaborate Network (RSCN) of the EIP on AHA

The RSCN brings together all EIP on AHA Reference Sites, and Candidate Reference Sites, across Europe into a single forum. The aim is to promote cooperation and develop and promote areas of innovative good practice and solutions, which contribute to improved health and care outcomes for citizens across Europe. The hope is to develop sustainable economic growth and create jobs. Members of 13 EIP on AHA Reference Sites (2013) have agreed on the AIRWAYS ICPs concept and are co-authors of the paper published in Clinical Translational Allergy [15]. A meeting of all EIP on AHA Reference Sites was co-organised by the Région LR, North England [85] and the EIP on AHA Reference Site Collaborative Network to scale up AIRWAYS ICPs in all Reference Sites (October 21, 2014). 74 EIP on AHA Reference Sites have now been approved by the EU (2016). A Twinning project has also been approved by the EIP on AHA to deploy MASK in 13 Reference Sites in order to compare allergic rhinitis diagnoses by allergists in adults and older people to study phenotypes, treatments and care pathways of rhinitis.

## Conclusion

 ARIA has evolved from a rigorously developed guideline to a mobile technology-based implementation strategy in order to provide an active and healthy life to rhinitis sufferers, whatever their age, sex or socio-economic status with the aim to reduce health and social inequalities incurred by this very common disease globally.

## Abbreviations

AIRWAYS ICPs: integrated care pathways for airway diseases
AR: allergic rhinitis
ARIA: Allergic Rhinitis and its Impact on Asthma
CDSS: Clinical Decision Support System
COPD: chronic obstructive pulmonary disease
DG: Directorate General
EAACI: European Academy of Allergy and Clinical Immunology
EIP on AHA: European Innovation Partnership on Active and Healthy Ageing
EU: European Union
FP: Framework Programme (EU)
GA^2^LEN: Global Allergy and Asthma European Network (FP6)
GARD: WHO Global Alliance against Chronic Respiratory Diseases
GRADE: Grading of Recommendation, Assessment, Development and Evaluation
HCP: health care professional
ICP: integrated care pathway
IPCRG: International Primary Care Respiratory Group
MACVIA-LR: Contre les Maladies Chroniques pour un Vieillissement Actif (fighting chronic diseases for active and healthy ageing)
MASK: MACVIA-ARIA Sentinel NetworK
MeDALL: Mechanisms of the Development of ALLergy (EU FP7)
MOH: Ministry of Health
NCD: non-communicable disease
NHS: National Health Service
RCT: randomized controlled trial
RQLQ: Rhinoconjunctivitis Quality of Life Questionnaire
RSCN: Reference Site Collaborative Network
SCUAD: severe chronic upper airway disease
VAS: visual analogue scale
WHO: World Health Organization
