# Correction to: ARIA 2016: Care pathways implementing emerging technologies for predictive medicine in rhinitis and asthma across the life cycle

**DOI:** 10.1186/s13601-019-0289-0

**Published:** 2019-10-10

**Authors:** J. Bousquet, M. van Hage

**Affiliations:** 1 Montpellier University Hospital, Montpellier, France; 2 MACVIA-France, Contre les MAladies Chroniques pour un VIeillissement Actif en France, European Innovation Partnership on Active and Healthy Ageing Reference Site, Montpellier, France; 3 INSERM, U1168, Ageing and Chronic Diseases Epidemiological and Public Health Approaches, 94800 Villejuif, France; 4 CHRU Arnaud de Villeneuve, 371 Avenue du Doyen Gaston Giraud, 34295 Montpellier Cedex 5, France; 5 Clinical Immunology and Allergy Unit, Department of Medicine Solna, Karolinska Institutet, Stockholm, Sweden; 6 Karolinska University Hospital, Stockholm, Sweden

## Correction to: Clin Transl Allergy (2016) 6:47 10.1186/s13601-016-0137-4

Following publication of the original article [1], the authors reported that one of the authors’ names was spelled incorrectly. In this Correction the incorrect and correct author name are shown. In the author list of this Correction article, only the correct name of the affected author and the corresponding author are presented.

Originally the author name was published as:

- M. van Hague.

The correct author name is:

- M. van Hage.
